# Chemical composition and microbial quality of *Datshi and Zoety*, unripen cottage cheese of Bhutan

**DOI:** 10.1002/fsn3.2715

**Published:** 2022-04-21

**Authors:** Anooja Nair, Dechen Choden, Monika Pradhan

**Affiliations:** ^1^ Department of Food Science and Technology College of Natural Resources Royal University of Bhutan Lobesa Bhutan

**Keywords:** ash, cottage cheese, Datshi, fat, microbes, moisture

## Abstract

*Datshi* is an unripen traditional Bhutanese cottage cheese produced commonly from the cow milk. It is consumed in two forms: fresh (*Datshi*) and matured Datshi (*Zoety*). The chemical and microbial compositions of both *Datshi* *and* *Zoety* were investigated in this study. The results showed that both *Datshi* *and* *Zoety* contain high moisture: 68.7% and 73.7%, respectively. However, *Datshi* contains good amount of protein, fat, and ash: 30.1%, 6.6%, and 6.9%, respectively; and in comparison to *Zoety*: protein (27.7%), fat (3.6%), and ash (2.1%). Regarding the microbial load, total aerobes and yeast and mold in Datshi are 10.5 log cfu/g and 8.3 log cfu/g, and that in Zoety is 11.3 log cfu/g and 9 log cfu/g, respectively. This study clearly demonstrated that chemical and microbial composition changes significantly when Datshi is transformed into Zoety, especially the chemical composition decreases significantly in Zoety. However, to understand the role of microbes in this transformation, further study is required for the identification of microflora of Datshi as well as Zoety.

## Graphical abstract

The chemical and microbial composition changes significantly when datshi is transformed into zeoty especially fat %, protein % and ash %. The variation in microbial composition was also noticed between the products, however further study is required to understand the role of microbes in the transformation.

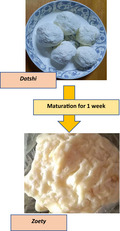

## 1 INTRODUCTION

**FIGURE 1:**
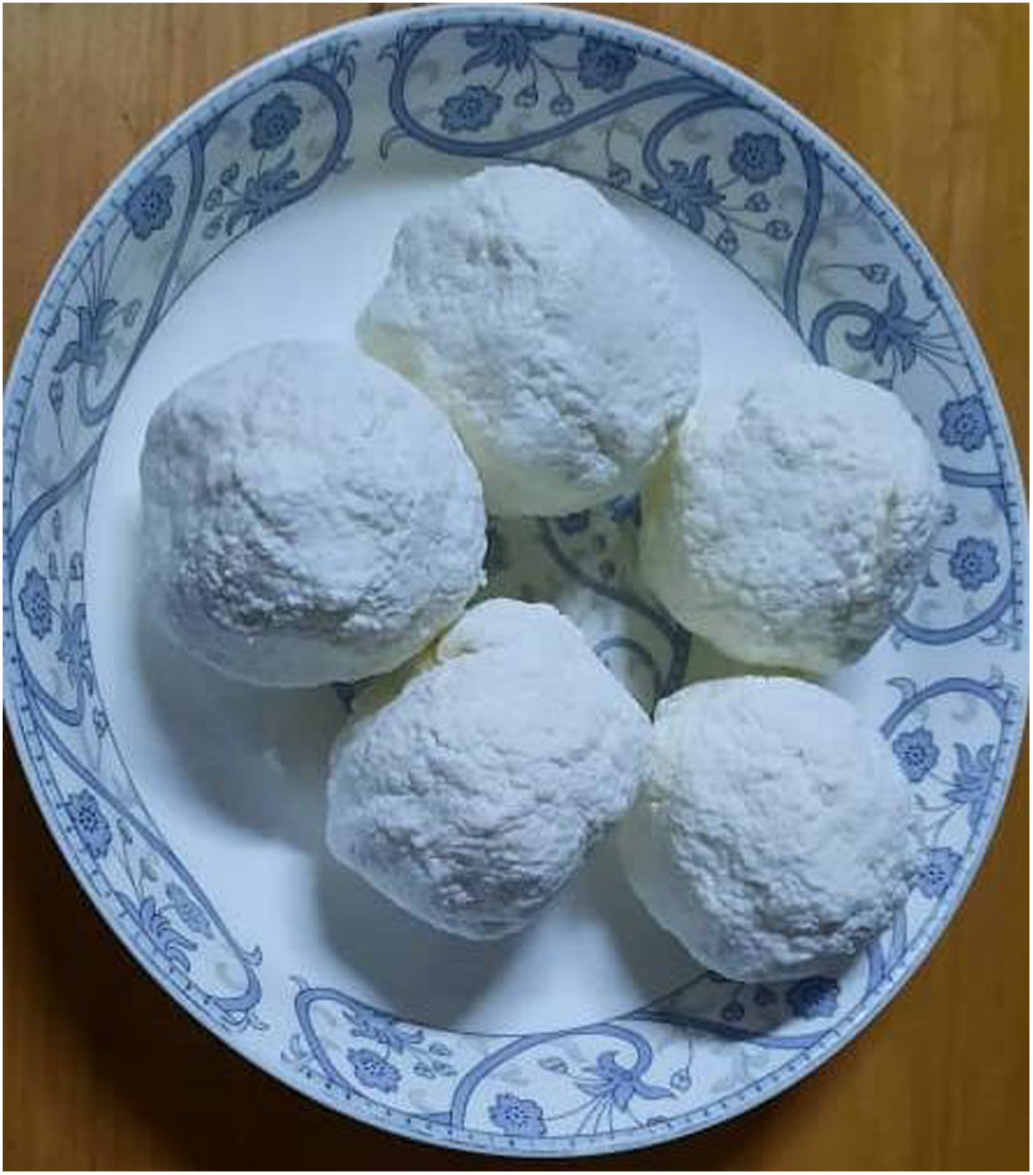
Picture of Datshi made into balls for handling

Datshi is a type of cottage cheese in Bhutan that is traditionally made using an indigenous process and is commonly made from cow's milk. It is a naturally fermented milk product made using the “back‐slopping” process, which involves inoculating the new batch with the previous batch's fermented milk product (Tamang et al., 2016). It is a partially defatted cheese made from fermented milk that has been churned to remove the fat as butter. It is made from buttermilk that has been gently heated over an open flame. The heating causes caseins to coagulate, which is then collected and squeezed to eliminate extra whey before being rolled into appropriate‐sized cheese balls (BAFRA, 2017; Rai et al., 2016). The texture is comparable to cottage cheese, with minute granules of coagulated casein and a mild flavor (Figure 1); however, unlike other cottage cheeses, it does not contain additional salt (Pozzobon & Pozzobon, 2019).

Datshi is widely produced and consumed on a daily basis (Shangpliang et al., 2017). Bhutanese of all ages enjoy it, and it holds a particular position in Bhutanese cuisine. The name of the cheese used in the famous Bhutanese cuisine “emadatsi,” which is a curry with chiles and cheese, and comes from the cheese used (Rai et al., 2016). Bhutan produced 3953 metric tons of Datshi in 2018 (DOL, 2018), which was used domestically. Bhutan currently does not export its cheese since local demand outweighs supply (Joshi & Gurung, 2009). The majority of the milk produced is turned into butter and cheese, which are staples in Bhutanese cuisine (Joshi & Gurung, 2009). According to the Department of Livestock (DOL) figures, 74% of the milk produced in 2018 was utilized in the production of dairy products, while the rest was consumed or sold as liquid milk (DOL, 2018). According to the same research, the main dairy products generated from the milk were butter, Datshi (unripe cottage cheese), and Chugo (hard cheese), with Datshi being the most common dairy item produced, followed by butter and Chugo.

**FIGURE 2:**
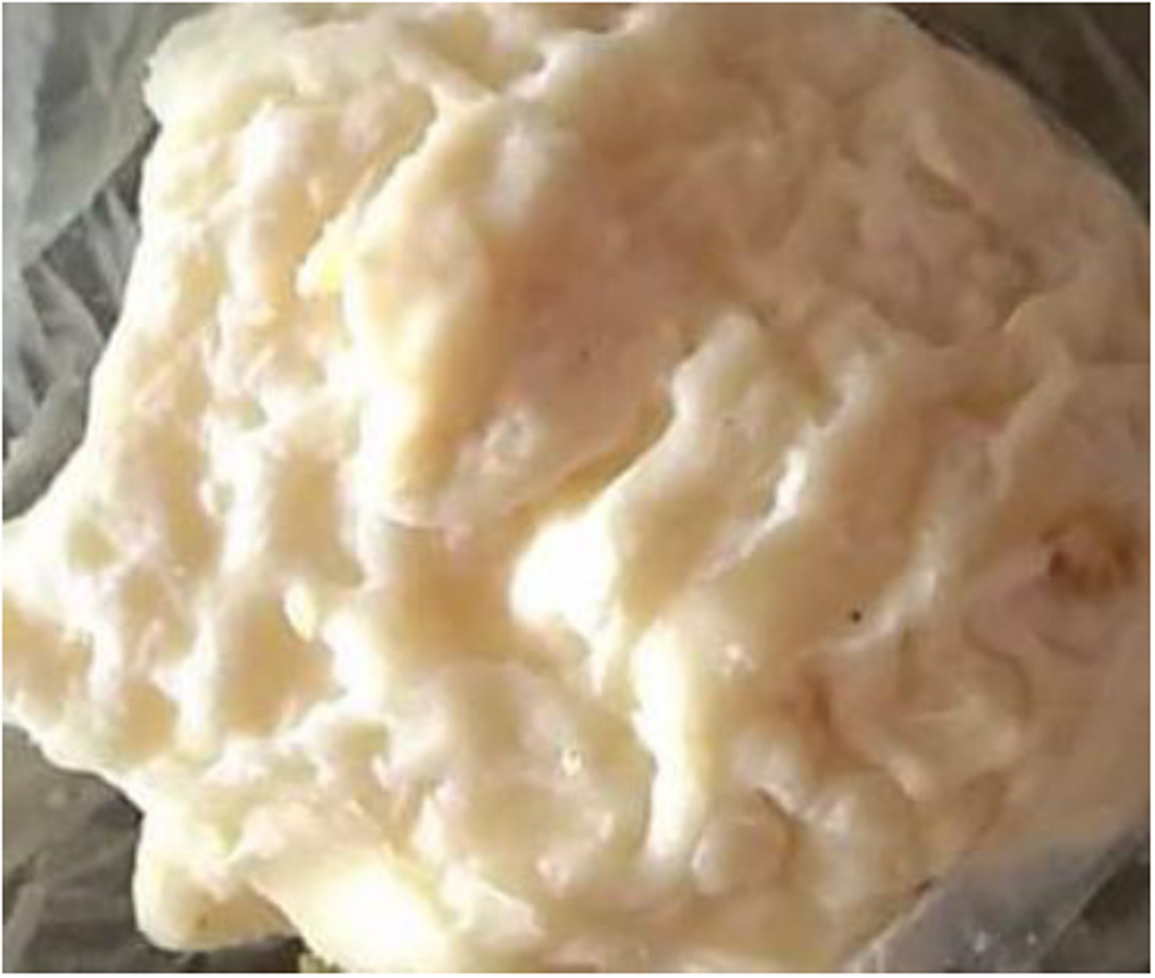
Picture of Zoety, mature Datshi. The Datshi changes into slimy gelatinous and has a strong smell

Matured Datshi, also known as Zoety, is another form of cottage cheese made by storing the fresh Datshi in loosely covered containers/plastic or tree leave wraps at room temperature. The product develops a pungent odor and a slimy, gelatinous exterior layer within a few days of storage, and is known as Zoety, as illustrated in Figure 2. Among many, it may be thought of as "spoiled cottage cheese," as described by Bodyfelt and Potter (2008), but for Bhutanese, it is a delicacy, and the small amount of gelatinous Zoety added to the curry is thought to enhance the dish's taste and flavor.

Despite the fact that *Datshi and Zoety* are frequently consumed by Bhutanese of all ages and contribute significantly to the Bhutanese diet, there is little information on its composition and microbial quality. As a result, the aim of this research was to determine the chemical and microbial compositions of *Datshi and Zoety*.

## 2 MATERIALS AND PROCEDURES

### 2.1 Sampling and processing of samples

Datshi samples were taken from Punakha Dzongkhag's Khuruthang Sunday vegetable market. This location was chosen because vendors from different Dzongkhags come to sell their agriculture produce including Datshi. Since there is no standard technique of preparation for Datshi because it is made by small‐scale dairy producers, 24 samples each weighing 300 gm were collected as representative samples from the indicated location. The samples were packed in a sterile plastic bag and transferred to Science Lab of College of Natural Resources, Lobesa, which is under Punakha Dzongkhag. The cheese samples were kept refrigerated until the next day, when they were analyzed.

### 2.2 Preparation of the sample

The composite samples were made from the 24 samples collected, from which two batches of samples were prepared consisting of 12 samples for each batch; the first batch of samples was used right away for microbial and chemical composition analysis and the second batch of samples was stored at room temperature for 1 week to mature in loosely closed plastic bags. This was done specifically to imitate the household storage conditions that are widespread in Bhutan to make Zoety from Datshi. Microbial and chemical compositional analysis of Zoety was carried out after 1 week of storage.

In addition, Datshi was also prepared at the college laboratory under controlled condition.

### 2.3 Chemical composition analysis

#### 2.3.1 Moisture analysis

The moisture content of the cheese samples was evaluated by drying them in a force draft oven (Stainless Steel Memmert Type Hot Air Oven, Germany) at 70°C for 3 h. The moisture percentage was calculated as per Nielsen (2010) using the following formula used to compute the moisture content:

$$\text{Moisture}\;\%=\frac{\text{Weight of wet sample}‐\text{Weight of dry sample}}{\text{Weight of wet sample}}\times 100$$

#### 2.3.2 Ash analysis

The ash content of the cheese was determined using dry ashing method as mentioned by Nielsen (2010). In a muffle furnace (model‐EF3, Vecsrar), approximately 5 gm of sample was weighed in the crucible and heated at 450°C for 12–18 h. After removing the samples and cooling them in a desiccator, the ash percentage was estimated as follows:

$$\text{Ash}\%\frac{W_{2}‐W_{1}}{W}\times 100$$

W_2_ = Crucible weight + sample weight after 450°C ashing

W_1_ = Empty crucible weight (before ashing)

W = Wet sample weight

#### 2.3.3 Fat analysis

The fat percentage was determined by Soxhlet fat extraction method (Nielsen, 2010) using Soxhlet apparatus (SCS‐6, Hanna, India). Three grams of the sample was weighed and placed in the Soxhlet apparatus, where anhydrous ether with a boiling point of 70°C was used for extraction. The solvent was removed by heating for 15–20 min in a hot air oven and then putting it in a desecrator for approximately 5 min. The following formula was used to compute the fat percentage:

$$\text{Fat}\;\%=\frac{\text{gm of fat in the sample}}{\text{gm of sample}}\times 100$$

#### 2.3.4 Protein analysis

The total nitrogen content was determined by the Kjeldahl method (Nielsen, 2010) using the Kjeldahl apparatus (model—Classic Dx, Pelican company). The crude protein was calculated using a conversion factor of 6.38. (Seifu, 2013; Nielsen, 2010).

#### 2.3.5 pH

A benchtop pH meter was used to determine the pH of both fresh and matured cheese (Model 211 microprocessor, HANNA, USA). The pH of 10 g of material was tested by homogenizing it in a 1:10 ratio with sterile distilled water and stirring it for 5 min (Panda et al., 2016).

### 2.4 Microbiological examination

For microbial analysis, samples from *Datshi and Zoety* prepared in the laboratory under controlled conditions were also analyzed along with the samples from the market.

#### 2.4.1 Total plate count

The total plate count was enumerated using the pour plate method. A series of dilutions were prepared in Hi‐media's peptone bacteriological solution (PBS) after the homogenization of the sample. One milliliter of sample was plated on plate count agar (Hi‐media) on a Petri dish and incubated at 30°C for 72 h after its solidification (ISO, 2013).

#### 2.4.2 Molds and yeast

Yeast and mold were inoculated in potato dextrose agar, and counted after incubating the plates at 28°C for 72 h (Himedia) (Panda et al., 2016).

### 2.5 Statistical analysis

All the laboratory experiments were carried out five times and the values are presented as mean ± standard deviation.

## 3 RESULTS AND DISCUSSION

### 3.1 A step‐wise procedure for the preparation of Datshi in controlled condition

#### 3.1.1 Heating of milk

**FIGURE 3:**
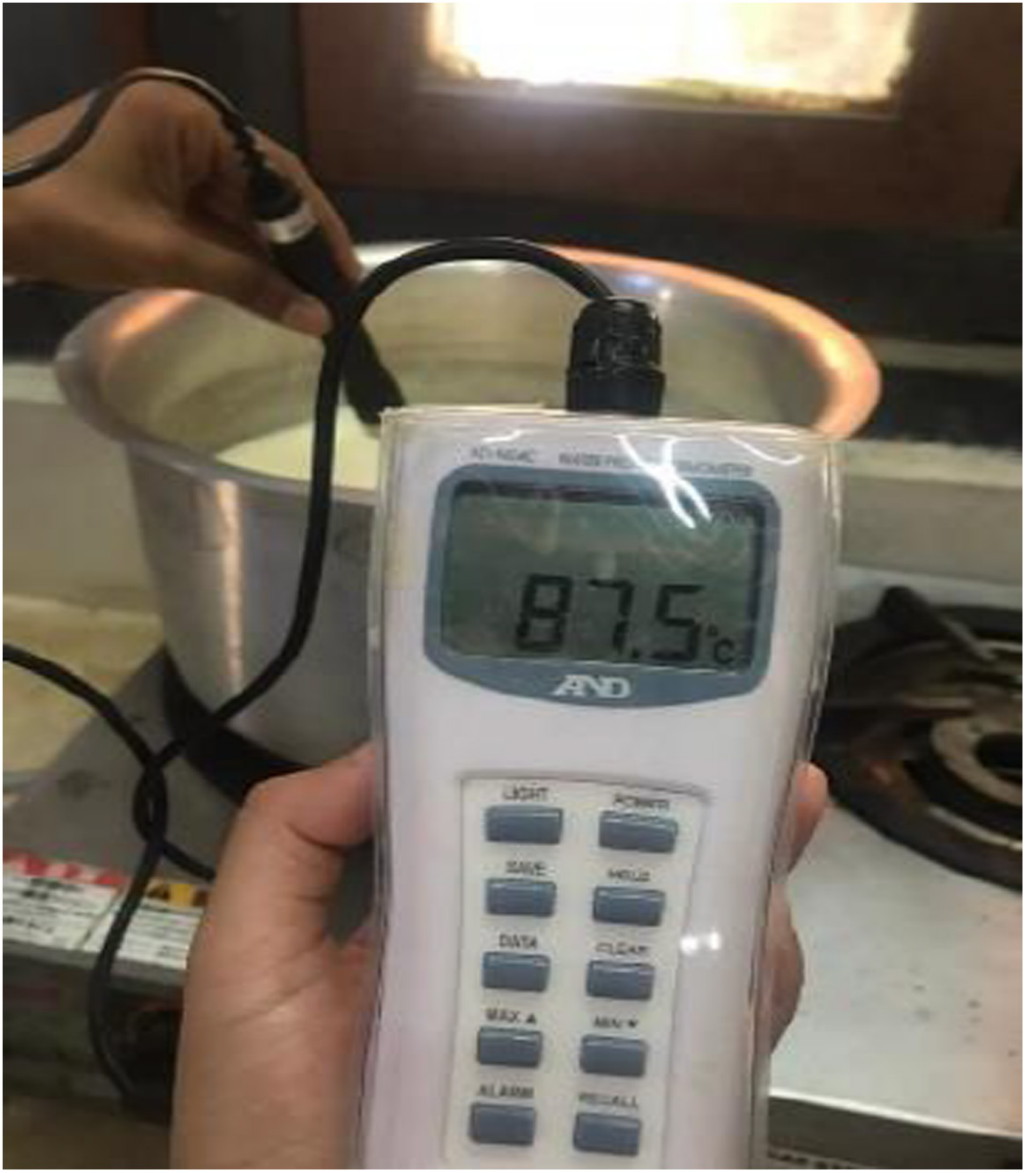
Boiling of milk

To kill the pathogenic bacterium, cow milk is boiled at 87.5°C for 10 min (Figure 3) and then allowed to cool at room temperature. The thermometer was used to record the temperature.

#### 3.1.2 Addition of starter culture and fermentation

**FIGURE 4:**
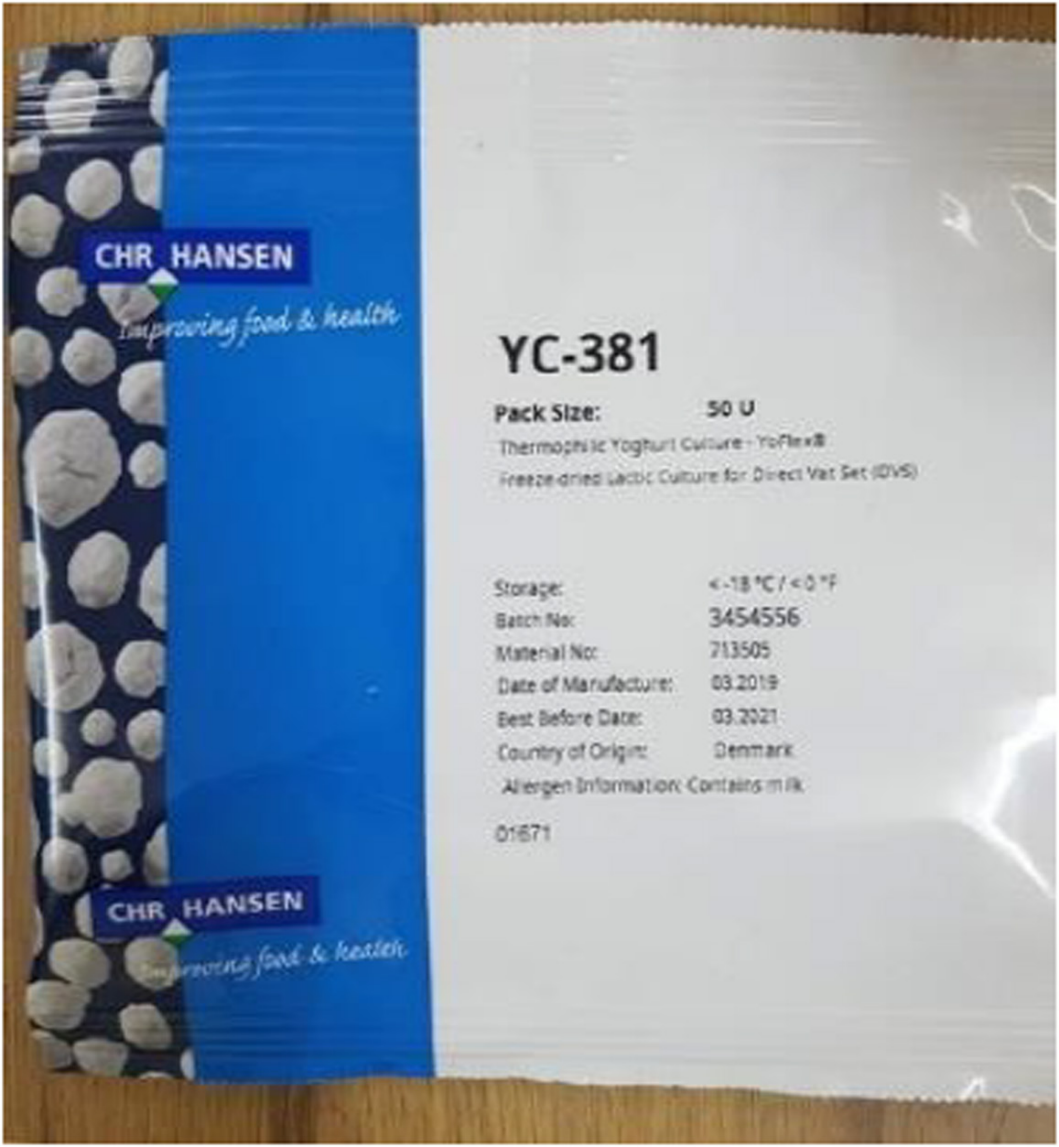
Starter culture used

**FIGURE 5:**
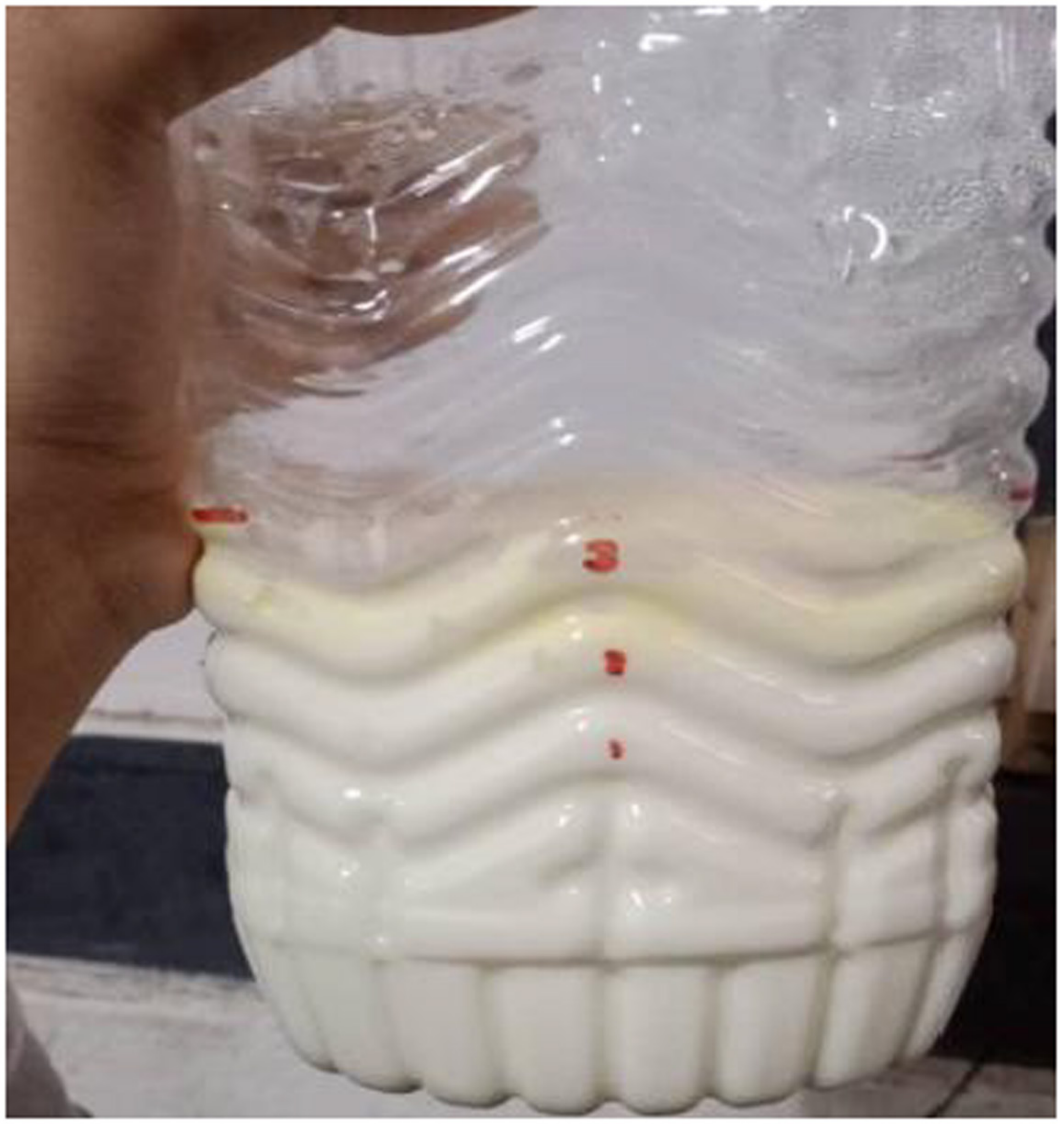
Fermented milk

For fermentation, the starter culture (thermophilic yogurt culture, CHR Hansen YF‐L811) was added, which is a specified mixed‐strain culture containing *Streptococcus thermophilus* and *Lactobacilus delbrueckii subsp. bulgaricus*. This starter culture is intended for the production of yogurt; instead, it was utilized to make Datshi because other starter cultures for the production of cheeses were unavailable. The starter cultures were introduced to the milk at a rate of 0.1% of the total volume (Figure 4). Following the addition of the starter culture, the milk was stored at 35°C in a clean container and fermented for 20 h (Figure 5). In the conventional process, fermentation is started by back‐slopping, which involves adding a little amount of curd from a previous batch (Panda et al., 2016). The curd was churned after 20 h of fermentation to separate the butter.

#### 3.1.3 Precipitation and boiling of butter milk

**FIGURE 6:**
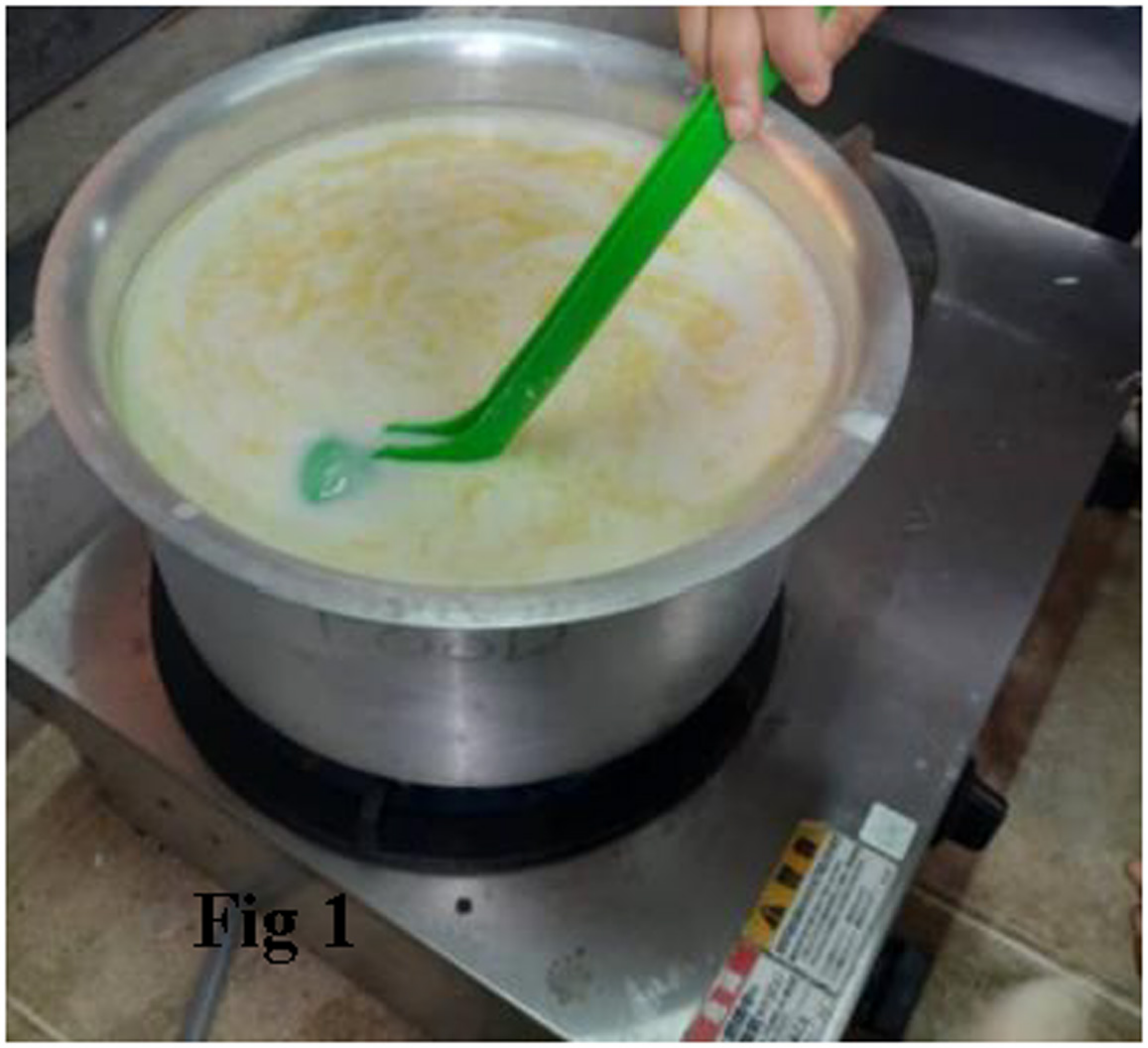
Heating of buttermilk and casein coagualtion

The butter was removed after separation, and the buttermilk was heated to 35–40°C. The precipitation of casein as a result of heating buttermilk is depicted in Figure 6.

#### 3.1.4 Coagulated casein straining and final product

**FIGURE 7:**
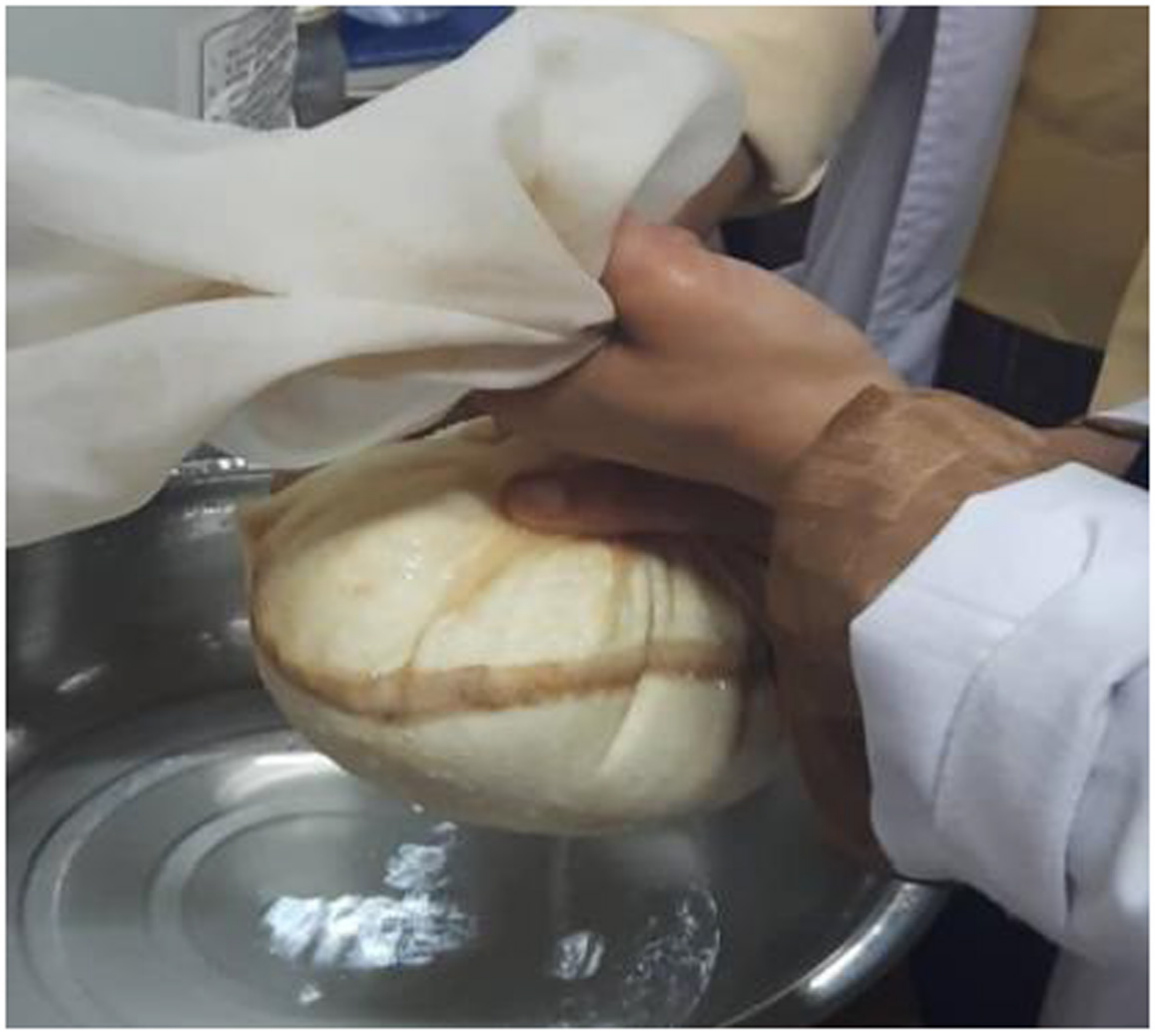
Straining of casein

**FIGURE 8:**
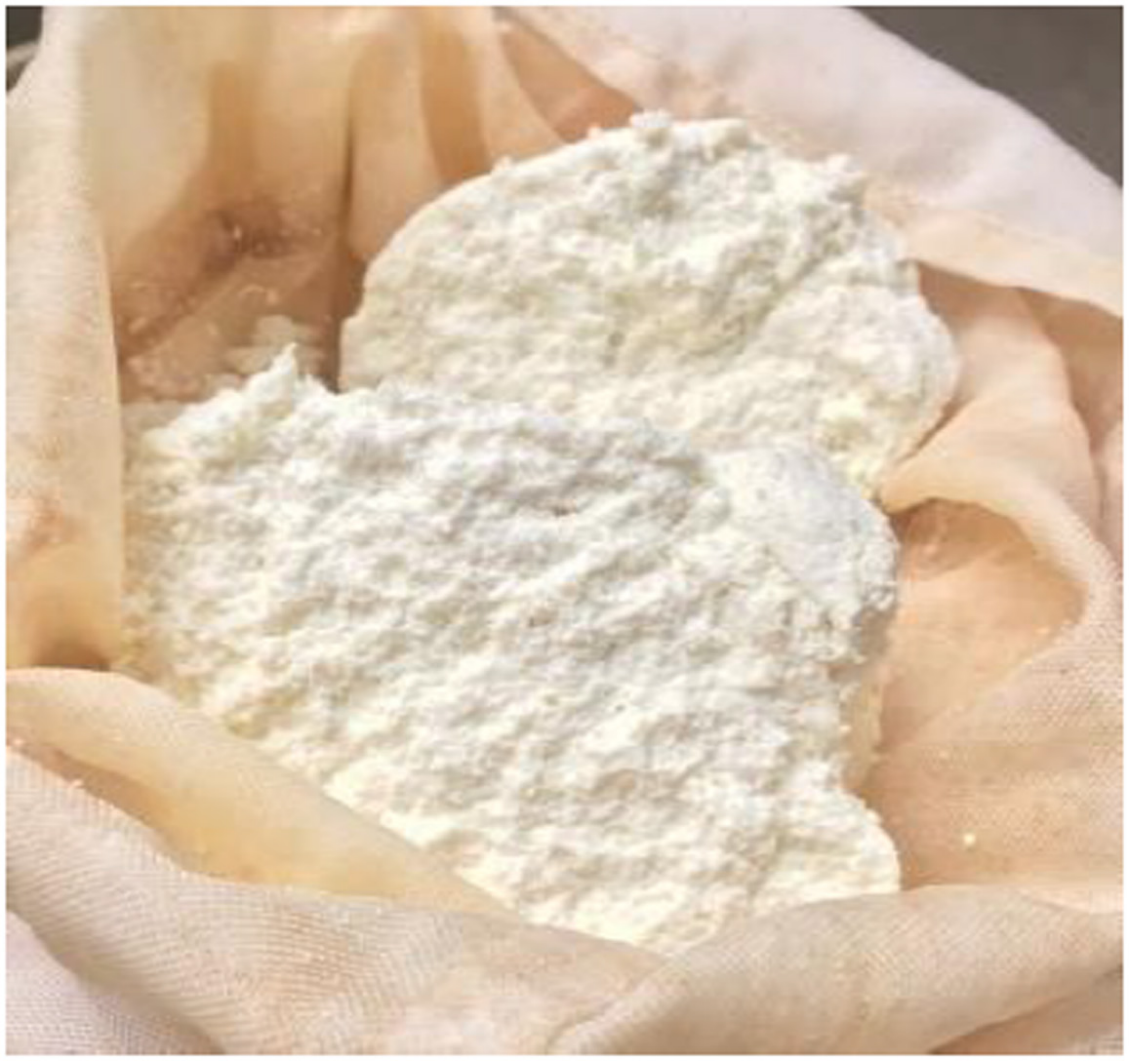
Datshi‐Final product

Next, the coagulated casein was strained using cheese cloth to eliminate any surplus water (Figure 7). To make it easier to use, the strained cheese (Figure 8) was rolled into smaller balls.

#### 3.1.5 Microbiological examination

Datshi has total aerobic counts of 10.5 log cfu/gm and yeast and mold counts of 8.3 log cfu/g. In Zoety, from market samples, the total aerobic count and yeast and mold were 11.3 log cfu/g and 9 log cfu/g, respectively. In case of *Datshi and Zoety* produced under controlled condition, the numbers of total aerobes and yeast and mold were 6 log cfu/g, 4 log cfu/g, 8 log cfu/g, and 7 log cfu/g, respectively. The microbial load in controlled samples was significantly lower than that of market samples. This could be because the degree of hygiene and sanitation maintained was relatively higher in case of controlled samples made in a laboratory which is not possible at the farm level without a standard method of processing.

#### 3.1.6 Chemical composition analysis

The moisture content of Datshi was 68.7%, as reported in Table 1. The moisture content of Datshi was lower than the moisture content of soft churpi as mentioned in the study conducted by Panda et al. (2016). Soft churpi is a fermented dairy product made from skim milk and is made in the same way as Datshi. The fat percentage is 6.6, which seems to be higher than the expected because it is supposed to have a lower fat content as a major part of fat is removed as butter after the churning. Similarly, the fresh Datshi examined had a protein level of 30.1% and an ash content of 6.9%. Datshi's increased crude protein and ash levels suggest that it could be a valuable source of amino acids and minerals for consumers (Seifu, 2013). Datshi has a pH of 5.02, indicating that the product is slightly acidic because it is made from fermented product (Panda et al., 2016).

**TABLE 1:** Proximate composition and acidity of *Datshi* *and* *Zoety*

Parameters	*Datshi*	*Zoety*
Moisture (%)	68.7933 ± 1.57	73.7892 ± 1.54
pH	5.0200 ± 0.76	6.6800 ± 0.59
Protein (%)	30.1417 ± 3.32	27.7625 ± 2.02
Fat (%)	6.6558 ± 1.85	3.6517 ± 0.90
Ash (%)	6.9500 ± 1.64	2.1817 ± 0.63

The experiments were repeated five times and the values are presented as Mean ± standard deviation.

In case of Zoety, the moisture content of 73.7% is significantly higher than that of Datshi. The increase in the moisture level of Zoety could be attributed to the free whey that was released during storage (Schmidt & Bouma, 1992). The ash content of Zoety (2.2%) was found to be significantly lower than that of Datshi (6.9%). The study's findings revealed that considerable demineralization occurred during the maturation of cheese. According to Prieto et al. (2002) and Lucey and Fox (1993), during the ripening of cheese, the phenomenon of demineralization occurs, which is caused by a reduction in pH, which further causes a certain solubilization of these minerals and their loss with the whey. The discrepancy could be due to the fact that the pH was not monitored every day. According to Prieto et al. (2002), a reduction in pH was noticed on the first week of fermentation, and on the seventh day of fermentation, the pH value of the cheese increased owing to the alkaline chemicals formed during the protein degradation that occurs during ripening. Thus, in the instance of Zoety, the drop in ash content could be attributed to a fall in pH during the first week of ripening; however, because the pH was only recorded on the 7th day, changes in pH on other days could not be reported. To get more insights into the demineralization, study on the influence of pH on the demineralization of Zoety can ascertain the phenomenon and also can give more information regarding the mineral content of Zoety.

The fat content of Zoety was significantly lower (3.6%) than that of Datshi. However, there is no significant difference found in the protein content and pH value between Zoety and Datshi.

## 4 CONCLUSION

According to the current study, the microbiological and chemical compositions of *Datshi and Zoety* are significantly different from one each other. The results revealed that hygiene and sanitation practices play a substantial effect in microbial load. As a result, standardization of processing and education of dairy farmers on sanitation and hygiene should be provided in order to create higher quality products. However, due to a lack of essential chemicals and reagents, chemical composition of *Datshi and Zoety* of laboratory samples could not be conducted. Because this is the first study of its kind on *Datshi and Zoety*, more research is needed to fully comprehend the involvement of bacteria and changes in chemical composition as Datshi transforms into Zoety.

## CONFLICT OF INTEREST

There is no conflict of interest.

## Data Availability

Data available on request from the authors.
